# Identifying predictors for food insecurity in England: a cross-sectional database analysis

**DOI:** 10.1186/s41043-025-00801-w

**Published:** 2025-02-28

**Authors:** Adam K. Stanley, Yasir Hadi, David Newbold, Hein Heuvelman, Anton Krige

**Affiliations:** 1 East Lancashire NHS Hospitals Trust, Blackburn, UK; 2 Edge Hill University, Liverpool, UK; 3 Faculty of Public Health, Blackburn with Darwen Borough Council, Blackburn, UK; 4 The University of Liverpool, Liverpool, UK; 5 University of Central Lancashire, Preston, UK

**Keywords:** Food security, Food poverty, Food insecurity, Nutrition, Inequality, Inequity

## Abstract

**Background:**

Nutrition plays a significant role in non-communicable disease worldwide and is a modifiable risk factor. Food security is defined as the ability of a household or individual to afford and access sufficient healthy and nutritious food. Food insecurity rates in the UK are among the worst in Europe and rising food prices have disproportionately affected lower income households. We aimed to identify predictors for food insecurity in England using nationally representative data.

**Methods:**

We conducted a database analysis on data collected in the ‘Food and You 2: Wave 6’ public cross-sectional dataset. Data were analysed from a mixed survey, collating 3,033 responses to the United Stated Department of Agriculture Household Food Security Survey Module, which defined food security status. We calculated risk ratios (RR) for food insecurity in relation to each independent variable, including sex, respondent age group, household size, presence of children in household, income, employment status, urban/rural living status, ethnicity, chronic conditions and Index of Multiple Deprivation (IMD).

**Results:**

72.3% (*n* = 2,194) were food secure, 23.4% (*n* = 710) were food-insecure. Variables associated with increased food insecurity risk included all respondent age groups below 65 year old, household size of 5 or more, presence of children under 16 years and under 6 years, household income less than £64,000 per annum, unemployed individuals, students, Asian / Asian British and African / African British ethnicities, presence of one or more chronic conditions and IMD of 1.

**Conclusions:**

In this analysis of nationally representative data, age, household size, presence of children, income, employment status, ethnicity and IMD were all associated with significantly increased risk for food insecurity. Further work is required to understand the relationship between these variables and food security in order to develop screening tools to identify those at highest risk of food insecurity in the population. This will help facilitate the effective provision of support to those who need it the most.

## Background

Nutrition is a primary factor in many non-communicable chronic diseases and poor diet is the risk factor with the highest impact on the NHS budget [1, 2]. In the United States, 6 in 10 adults are living with a diet-related chronic disease [3]. Diet represents a significant factor in the aetiology of non-communicable diseases, including cancer, diabetes mellitus and ischaemic heart disease [4]. The World Health Organisation (WHO) has defined diet as one of the 4 main modifiable risk factors in the aetiology of chronic disease [5]. Research also suggests that nutrition-related chronic disease results in significant long-term productivity and financial costs. Productivity losses are estimated at around 10% of lifetime earnings, while gross domestic product (GDP) losses resulting from undernutrition are estimated to be between 2–3% [6]. Nutrition has also been linked to cognitive development and educational outcomes [6].

### Food security

The United Kingdom Food Security Report (2021) defined household food security as the ability to ‘reliably afford and access sufficient healthy and nutritious food’ [7]. Data have demonstrated the disproportionate effect of rising food prices on lower income households. Between 2006 and 2020, the average UK household spent between 10–12% of their income on food and non-alcoholic drinks, whereas those in the lowest quintile by equivalised disposable income spent between 14–17% of their income on food and non-alcoholic drinks [7]. In 2006, the United States Department of Agriculture (USDA) revised the wording of their definitions for food security, including ‘High’, ‘Marginal’, ‘Low’ and ‘Very Low’ food security [8].

### USDA household food security survey module (HFSSM)

The USDA Food and Nutrition Service (FNS) began developing the Current Population Survey (CPS) Food Security Supplement in 1996, alongside the National Centre for Health Statistics and Mathematica Policy Research, Inc. (MPR) [9]. Rasch Modelling was used to assign statistical ‘severity levels’ to each of the questions contained in the food security survey in order to produce a continuous food security measure, which allowed for categorisation of food security status based on survey responses. The stability of this model over time was tested by estimating the model independently on three separate CPS datasets between 1995–1997. The model was also estimated independently across different population subgroups, stratified by race/ethnicity, household composition, metropolitan status and country region, finding good consistency across subgroups. Annual reports including the Food Security Supplement data have been published since 1999. Since then, multiple validation studies have been published [10–13]. Frongillo found the model to provide valid measurement of food security at both the household and individual level [14]. Validation studies have been reported in Latin America and the Caribbean, which suggest food security as reported by the HFSSM is significantly associated with intake of micronutrient-rich foods, as well as dietary variety [15]. Validation studies remain scarce in the United Kingdom, although data suggests HFSSM food insecurity is associated with reduced fruit and vegetable intake [16]. As such, the HFSSM has become widely accepted as a valid measurement for Food Security, leading to its incorporation into national health surveys.

Food insecurity rates in the UK are among the worst in Europe and give rise to significant physical and mental health burdens [17]. In the most recent UK Food Security Report, 8% of households were reported as being food-insecure with 4% reporting ‘very low’ food security [7]. The use of emergency food parcels rose by 120% across England in the last 6 years [18]. In the North West of England, there was only a rise of 75%. However, this region has some of the highest rates of food insecurity in the country [19]. This may suggest potential disparities in the provision of food support to those at greatest need. It is therefore essential to employ more proactive approaches to the provision of food support. To achieve this, we must be able to accurately screen for those at high risk of food insecurity amongst the population. It is therefore imperative to have a detailed understanding of the sociodemographic factors associated with food insecurity at the household level. Various factors have been suggested to be associated with food insecurity in the USA, including household income, presence of children, race and more [20]. Understanding the risk relationship between these variables and food insecurity could allow for risk screening initiatives, in order to connect households at risk with appropriate support services, via social prescribing practices [21, 22]. We aimed to evaluate aggregate level data to identify factors associated with household food insecurity in a UK setting.

## Methods

We conducted a cross-sectional database analysis using publicly available data from the ‘Food and You 2: Wave 6’ Government survey. This is a biannual mixed-mode (online and postal) survey which collects information on self-reported consumer food safety behaviours amongst adults in England, aged 16 years old and over [23]. Participant addresses were randomly selected from the Royal Mail’s Postcode Address File. Data were collected between 12th October 2022 and 10th January 2023. The food security data analysed were contained within Tables 1162, 1163, 1164, 1165 and 1166 of the ‘Food and You 2: Wave 6’ public data tables, pertaining to the England data. Data were weighted to compensate for (i) variations in sample selection probabilities and propensities to respond within households and (ii) response rate variations by country, region, age and sex profile, and local level of deprivation. The primary outcome was food security, as defined by the USDA 10-item HFSSM [8]. This is a standardised measure of food security experienced at the household level over the previous 12 months (Appendix 1). The module was distributed to all participants. Due to the sensitive nature of variables collected, responses included ‘Prefer not to say’, ‘Don’t know’ or ‘Not stated’ options. Data were stratified by each independent variable and compared between the Food-Secure group (those with ‘High’ or ‘Marginal’ food security) and the Food-Insecure group (those with ‘Low’ or ‘Very low’ food security). The dependent variable was food security status. Independent variables included: Sex, Respondent age group (16–24, 25–34, 35–44, 45–54, 55–64, 65–79, 80+), Household size (1, 2, 3, 4, 5+), Children < 16 years old, Children < 6 years old, Household income per annum (<£19,000, £19,000–31,999, £32,000–63,999, £64,000–96,000, >£96,000), Employment status (Employed, Unemployed, Student), Urban/Rural living status, Ethnicity (White, Mixed, Asian / Asian British, African / African British), Chronic conditions and Index of Multiple Deprivation (IMD) [1–5]. The IMD is a relative measure of deprivation, which represents a combination of the seven domains of deprivation (income deprivation; employment deprivation; education, skills and training deprivation; health deprivation and disability; crime; barriers to housing and services and living environment deprivation), weighted according to the ‘English Indices of Deprivation 2019’ document [24]. Pearson’s Chi-squared tests of association were used to identify variables significantly associated with food security status and generate p values. We then calculated Risk Ratios (RR) for food insecurity in relation to each independent variable to compare their effect sizes.

Data were analysed from a total of 3,032 responses, collated in the ‘Food and You 2: Wave 6’ dataset. 61.2% were online responses (*n* = 1,855) and 38.8% paper responses (*n* = 1,177). Of those who stated, 48.7% were male (*n* = 1,451) and 51.3% female (*n* = 1,528). Variables investigated included Sex, Respondent age range, Household size, Presence of children < 16 years old, Presence of children < 6 years old, Household income range, Employment status, Urban / rural living status, Ethnicity, Presence of chronic condition and IMD.

**Fig. 1:**
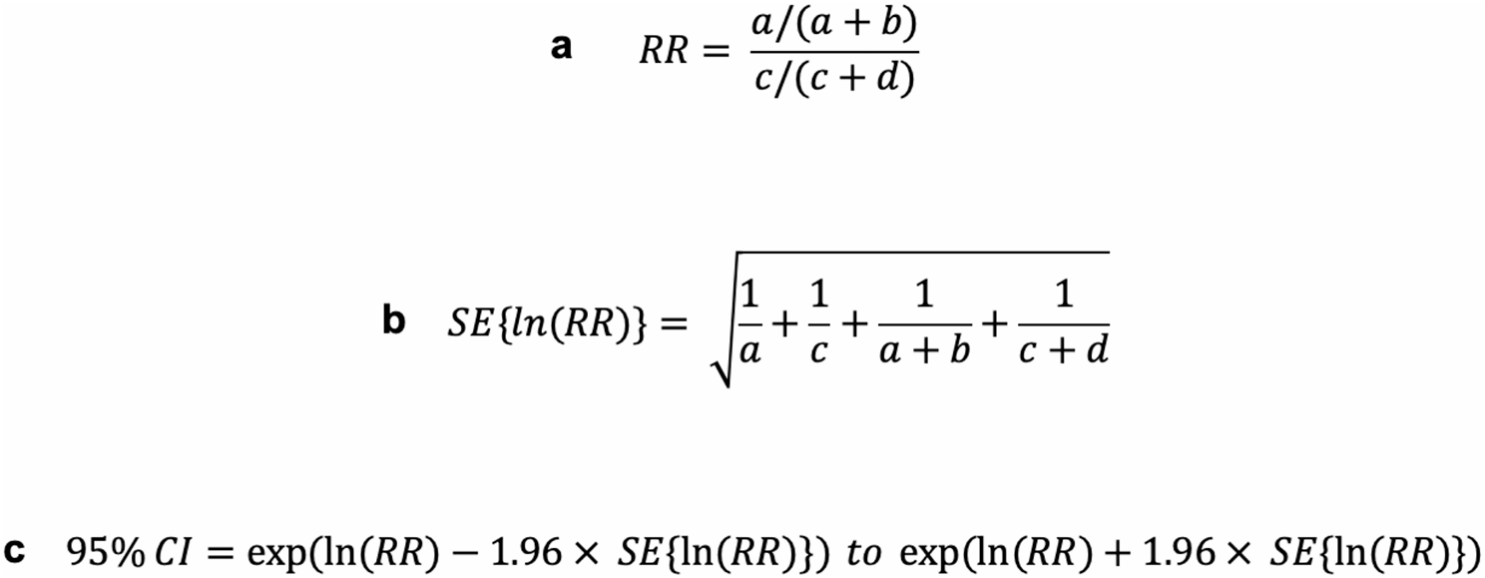
RR, SE and 95% CI formula

The relative risk or risk ratio (RR) was calculated as shown in Fig. 1a, where ‘a’ is the number of food-insecure households and ‘b’ is the number of food-secure households within the variable of interest, ‘c’ is the number of food insecure households in the reference variable (most food secure) and ‘d’ is the number of food-secure households in the reference variable. The Standard Error (SE) of the log relative risk was calculated as shown in Fig. 1b. 95% confidence intervals (95% CI) were calculated as shown in Fig. 1c.

We then constructed a forest plot to illustrate RR effect size of each variable on food security. Significantly associated variables were demonstrated by 95% confidence intervals which did not intersect the RR reference line at a value of 1.0 on the x axis. Statistical analysis and graph construction was conducted using IBM SPSS^®^ Statistics version 29.0.2.0 and Microsoft Excel^®^. RR values are reported as [RR (95% CI)]. All p values expressed related to Pearson’s Chi-Squared tests. The alpha level was set at 0.05.

## Results

Of those who responded, 72.4% (*n* = 2194) were classed as food secure (‘high’ or ‘marginal’ food security). 23.4% (*n* = 710) were classed as food-insecure (‘low’ or ‘very low’ food security). Food security was ‘not stated’ in 4.2% (*n* = 128). Respondent characteristics are detailed in Table 1, compared between the food secure and food insecure groups.

**Table 1:** Respondent characteristics, compared between food-secure and food-insecure groups, data included for those where food security status was available based on collected data

Variable	All	Food insecure	Food secure	*P* value
Sex				0.017
Male	1386	307	1079	
Female	1476	383	1093	
Age				< 0.001
16–24	346	152	194	
25–34	471	173	297	
35–44	460	142	318	
45–54	483	105	378	
55–64	459	75	385	
65–79	494	44	450	
80+	168	12	156	
Household size				< 0.001
1	335	72	263	
2	1115	181	934	
3	548	147	401	
4	462	123	339	
5+	332	149	183	
Children < 16				< 0.001
Yes	855	312	543	
No	1975	377	1598	
Children < 6				< 0.001
Yes	351	158	193	
No	2455	526	1929	
Household income (£)				< 0.001
< 19,000	554	257	297	
19,000–31,999	513	165	349	
32,000–63,999	666	117	548	
64,000–96,000	277	15	262	
> 96,000	164	6	158	
Employment				< 0.001
Employed	2540	570	1970	
Unemployed	89	47	42	
Student	200	75	125	
Urban / rural				< 0.001
Urban	2375	630	1745	
Rural	529	80	449	
Ethnicity				< 0.001
White	2405	532	1873	
Mixed	51	17	34	
Asian / Asian british	216	75	141	
African / African British	99	38	61	
Chronic conditions				< 0.001
Yes	804	251	553	
No	1899	387	1512	
IMD				< 0.001
1 (Most Dep)	567	226	341	
2	585	152	433	
3	583	162	421	
4	589	106	483	
5 (Least Dep)	579	64	515	

**Fig. 2:**
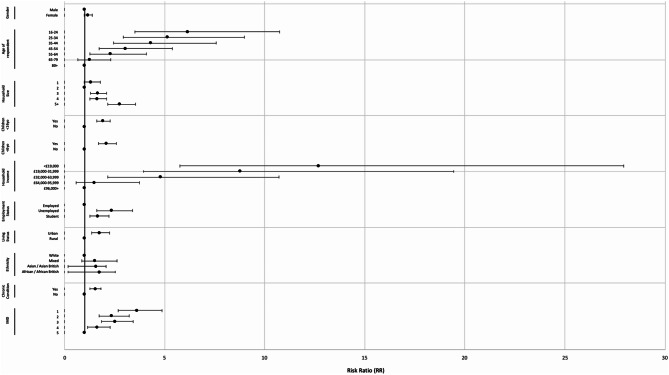
Forest plot of Risk Ratio of food insecurity (RR) by variable

Chi-squared tests revealed significant associations between all variables investigated and food insecurity status. P values are detailed in Tables 1, 2 shows the RR of food insecurity, according to each variable investigated. Sex was not significantly associated with an increase or decrease in RR of food security. Respondent age was inversely associated with food insecurity. Compared with 80 + year olds, the relative risk of food insecurity was greater the younger the age group, with 16–24 year-olds most at risk [RR 6.15 (3.52–10.75)]. Household size of 2 was the most food secure. Household size of 5 + was associated with the greatest risk [RR 2.76 (2.16–3.55)]. Presence of children < 16 years and < 6 years were both associated with increased food insecurity risk [RR 1.91 (1.61–2.27) and RR 2.10 (1.70–2.59), respectively]. Household incomes of less than £19,000, £19,000–31,999 and 32,000–63,999 were associated with increased food insecurity risk [RR 12.68 (5.75–27.95), RR 8.77 (3.96–19.44) and RR 4.81 (2.16–10.73) respectively]. Both unemployed individuals [RR 2.35 (1.63–3.39)] and students [RR 1.67 (1.26–2.21)] had an increased risk. Urban living status conferred an increased risk [RR 1.75 (1.36–2.25)]. Asian / Asian British and African / African British ethnicities were associated with increased food insecurity risk [RR 1.57 (1.19–2.07) and RR 1.74 (1.18–2.55), respectively]. Presence of one or more chronic conditions was associated with increased risk [RR 1.53 (1.28–1.83)]. The most deprived quintile by IMD was associated with the greatest food insecurity risk [RR 3.61 (2.67–4.87)] compared to the least deprived quintile. The RR values are illustrated in Fig. 2.

**Table 2:** Food insecurity risk ratios (RR), standard error in RR (SE) and 95% confidence intervals (CI), lower bound (LB) and upper bound (UB)

Variable	Risk ratio (RR)	Standard error {in (RR)}	LB 95% CI	UP 95% CI
Sex				
Male	1.00			
Female	1.17	0.09	0.99	1.38
Age				
16–24	6.15	0.28	3.52	10.75
25–34	5.15	0.28	2.95	9.00
35–44	4.32	0.29	2.46	7.58
45–54	3.04	0.29	1.72	5.39
55–64	2.28	0.30	1.27	4.09
65–79	1.25	0.31	0.67	2.30
80+	1.00			
Household size				
1	1.32	0.15	0.98	1.79
2	1.00			
3	1.65	0.12	1.30	2.10
4	1.64	0.13	1.27	2.11
5+	2.76	0.13	2.16	3.55
Children < 16				
Yes	1.91	0.09	1.61	2.27
No	1.00			
Children < 6				
Yes	2.10	0.11	1.70	2.59
No	1.00			
Household income (£)				
< 19,000	12.68	0.40	5.75	27.95
19,000–31,999	8.77	0.41	3.96	19.44
32,000–63,999	4.81	0.41	2.16	10.73
64,000–96,000	1.48	0.47	0.59	3.74
> 96,000	1.00			
Employment				
Employed	1.00			
Unemployed	2.35	0.19	1.63	3.39
Student	1.67	0.14	1.26	2.21
Urban / rural				
Urban	1.75	0.13	1.36	2.25
Rural	1.00			
Ethnicity				
White	1.00			
Mixed	1.51	0.28	0.86	2.63
Asian / Asian British	1.57	0.14	1.19	2.07
African / African British	1.74	0.20	1.18	2.55
Chronic conditions				
Yes	1.53	0.09	1.28	1.83
No	1.00			
IMD				
1 (Most Dep)	3.61	0.15	2.67	4.87
2	2.35	0.16	1.72	3.22
3	2.51	0.16	1.84	3.43
4	1.63	0.17	1.17	2.27
5 (Least Dep)	1.00			

## Discussion

Our results demonstrate a significantly increased risk of food insecurity among younger responders, particularly those aged 16–24 years old, people with household size of 5 or more, people in a household with children less than 16 years old, people with a household income less than £64,000 per annum, unemployed individuals, students, Asian or Asian-British individuals, African or African-British individuals and people with an IMD less than 5.

Our results reflect the wider health inequities observed in the literature [25–31]. Many of the factors investigated in this dataset have been recognised in the literature in the context of unequal access to healthcare and poor long-term health outcomes. Minoritised ethnic groups are at higher risk of multiple chronic conditions and have impaired access to primary care [28, 29]. It is also widely recognised that IMD is closely correlated with health outcomes. Individuals from lower IMD groups have generally poorer health outcomes [30, 31]. Furthermore, data indicates that inequalities in access and uptake of healthcare between different social classes have increased [32]. This relationship is evident in the context of food security, with more deprived households spending a larger proportion of their household income on food and non-alcoholic beverages [7].

It is challenging to draw any causal relationships on the multivariable level due to the complexity and interplay between sociodemographic factors in the context of health inequities. However, gaining an understanding of the associations between food insecurity and variables that are easily measurable and accessible in clinical practice can aid in the development of effective risk screening. As mentioned previously, this would allow for appropriate social referral pathways to be made use of, including social prescribers or link workers [21, 22].

### Strengths & limitations

This study used nationally representative data from Wave 6 of the Food Standards Agency (FSA) ‘Food and You 2’ official statistic survey, conducted by Ipsos and the FSA [33]. The large sample size allowed for effective subgroup analysis of the data and increased the generalisability of our findings. Participant addresses were randomly selected from the Royal Mail’s Postcode Address File. This helped in reducing selection bias or limitations associated with convenience sampling. Use of a mixed-mode survey, collecting both online and postal responses facilitated the capture of a wider respondent sample compared to unimodality. However, despite this, population groups with limitations to accessibility of online resources and postal participation may not have been represented appropriately in the sample. In addition, the online surveys contained built-in checks to ensure correct answer format, whereas postal surveys were unable to control or regulate the respondent’s answer formats. As such, this may have resulted in a higher rate of missing data from postal responses compared to online responses and a consequential under-representation of those responding via mail.

The survey made use of an internationally validated food security survey (HFSSM). Classification criteria for food security status therefore remained constant across the whole sample. This also provided a reliable and objective measure of food security status in our sample, generalisable to the wider food security literature. The use of relative risk analysis allowed not only for the determination of statistical significance, by way of 95% CIs, but also provided an objective evaluation of the effect size of each variable investigated. This allowed for comparison between food insecurity risk of different investigated variables, as illustrated in the forest plot in Fig. 2.

A limitation of the survey data collected included the self-reported outcome measures. This gave way to more subjective outcome measures, and potential for recall bias. However, in the classification of food security status, use of this internationally validated survey provided the most objective outcome measure possible to achieve using a survey-based data collection method.

Furthermore, the questions contained in the USDA HFSSM primarily focus on gathering data around food quantity and financial accessibility rather than specific measurements of food groups or nutrient intake. This may represent a limitation in the reliability of the HFSSM, due to the nature of malnutrition being a problem that incorporates obesity and excessive energy intake, without adequate nutritional value. As such, the measurement of macronutrient and micronutrient intake is required to fully assess whether an individual or household is adequately meeting their nutritional requirements. However, this may not be practical to measure at the national level. Additionally, extensive validation data exists and the literature does suggest correlation between HFSSM food security status and nutritional intake as discussed previously [15–18, 20, 21].

## Conclusions

We identified multiple significant predictors for food insecurity amongst the population, including age, household size, children, income, chronic conditions, ethnicity and IMD. While identifying risk factors for food insecurity amongst the population is an important first step, further work is required to understand the relationships between predictive variables and their effect size on food insecurity, in order to develop screening tools to identify those at risk of food insecurity, with optimal predictive value. In order to obtain more detailed understanding of predictor variables, a regression model and receiver operating characteristic (ROC) analysis is required to test the relationship between food security as a binary outcome and the multiple variables associated. Other research has also highlighted the importance of income and other financial-based measures, which seems to most accurately reflect food insecurity prevalence in the literature [34–36].

Following such analyses, we aim to construct an effective screening model based on local data, which can be used to identify those individuals at high-risk of food insecurity. Such individuals may then be referred to social prescribers, or link workers, who are well placed to advise on initial support pathways and contacts. This will provide a more proactive approach to the provision of food support services, which do not appear to be consistently delivered to at-need groups. Measures such as these are crucial in the effort to move ultimately from a largely reactive healthcare model to one more preventative in nature, which may yield improved long-term health outcomes and cost-effectiveness.

## Appendix 1: USDA household food security survey module

1. “We worried whether our food would run out before we got money to buy more.” Was that often, sometimes, or never true for you in the last 12 months?
2. “The food that we bought just didn’t last and we didn’t have money to get more.” Was that often, sometimes, or never true for you in the last 12 months?
3. “We couldn’t afford to eat balanced meals.” Was that often, sometimes, or never true for you in the last 12 months?
4. In the last 12 months, did you or other adults in the household ever cut the size of your meals or skip meals because there wasn’t enough money for food? (Yes/No)
5. (If yes to question 4) How often did this happen—almost every month, some months but not every month, or in only 1 or 2 months?
6. In the last 12 months, did you ever eat less than you felt you should because there wasn’t enough money for food? (Yes/No)
7. In the last 12 months, were you ever hungry, but didn’t eat, because there wasn’t enough money for food? (Yes/No)
8. In the last 12 months, did you lose weight because there wasn’t enough money for food? (Yes/No)
9. In the last 12 months did you or other adults in your household ever not eat for a whole day because there wasn’t enough money for food? (Yes/No)
10. (If yes to question 9) How often did this happen—almost every month, some months but not every month, or in only 1 or 2 months?

## Data Availability

The datasets generated and/or analysed during the current study are available in the United Kingdom Data Service (UKDS) repository, https://beta.ukdataservice.ac.uk/datacatalogue/studies/study?id=8814.

## Abbreviations

CI: Confidence Interval
CPS: Current Population Survey
FNS: Food and Nutrition Service
FSA: Food Standards Agency
GDP: Gross Domestic Product
HFSSM: Household Food Security Survey Module
IMD: Index of Multiple Deprivation
MPR: Mathematica Policy Research
ROC: Receiver Operating Characteristic
RR: Risk Ratio
SE: Standard Error
UK: United Kingdom
UKDS: United Kingdom Data Service
USA: United States of America
USDA: United States Department of Agriculture
WHO: World health Organisation
